# In vivo SPECT imaging of Tc-99m radiolabeled exosomes from human umbilical-cord derived mesenchymal stem cells in small animals

**DOI:** 10.1016/j.bj.2024.100721

**Published:** 2024-04-16

**Authors:** Yi-Hsiu Chung, Yi-Pei Ho, Shiou-Shiow Farn, Wei-Cheng Tsai, Zhi-Xiang Li, Tzou-Yien Lin, Chi-Chang Weng

**Affiliations:** a Department of Medical Research and Development, Research Division, Chang Gung Memorial Hospital at Linkou, Taoyuan, Taiwan; b Department of Medical Imaging and Radiological Sciences, Chang Gung University, Taoyuan, Taiwan; c Healthy Aging Research Center, Chang GungUniversity, Taoyuan, Taiwan; d Department of Isotope Application Research, National Atomic Research Institute, Taoyuan, Taiwan; e ExoOne Bio Co., Ltd., Taipei, Taiwan; f Department of Pediatrics, Division of Infectious Diseases, Chang Gung Memorial Hospital, Taoyuan, Taiwan; g College of Medicine, Chang Gung University, Taoyuan, Taiwan

**Keywords:** Tc-99m, UCMSC-EVs, SPECT, Biodistribution, Pharmacokinetic analyses

## Abstract

Extracellular vesicles derived from human umbilical cord-derived mesenchymal stem cells (UCMSC-EVs) have been postulated to have therapeutic potential for various diseases. However, the biodistribution and pharmacokinetics of these vesicles are still unclear. For a better understanding of the *in vivo* properties of UCMSC-EVs, in the present study, these vesicles were first radiolabeled with Technetium-99m (^99m^Tc-UCMSC-EVs) and evaluated using *in vivo* single photon emission computed tomography (SPECT) imaging and biodistribution experiments. SPECT images demonstrated that the liver and spleen tissues mainly took up the ^99m^Tc-UCMSC-EVs. The biodistribution study observed slight uptake in the thyroid and stomach, indicating that ^99m^Tc-UCMSC-EVs was stable at 24 h *in vivo*. The pharmacokinetic analyses of the blood half-life demonstrated the quick distribution phase (0.85 ± 0.28 min) and elimination phase (25.22 ± 20.76 min) in mice. This study provides a convenient and efficient method for ^99m^Tc-UCMSC-EVs preparation without disturbing their properties. In conclusion, the biodistribution, quick elimination, and suitable stability *in vivo* of ^99m^Tc-UCMSC-EVs were quantified by the noninvasive imaging and pharmacokinetic analyses, which provides useful information for indication selection, dosage protocol design, and toxicity assessment in future applications.

## Highlights

• The SPECT images demonstrated that ^99m^Tc-UCMSC-EVs were mainly taken up by the liver and spleen and slightly in the lungs, joints, and spine. Moreover, the accumulation of ^99m^Tc-UCMSC-EVs in the bladder implied that the kidneys could excrete this tracer. The pharmacokinetic analyses of the blood half-life demonstrated a 0.85 ± 0.28 min distribution phase and a 25.22 ± 20.76 min elimination phase. The upregulated expression of anti-CD73 in the spleen tissue corresponds to the autoradiogram image, which confirmed ^99m^Tc-UCMSC-EV accumulation.

## 1 Introduction

Exosomes are a type of membrane-bound extracellular vesicle (EV) produced in most eukaryotic cells' endosomal compartments [1]. Generally, exosomes are considered smaller than most other EVs and are approximately 30–150 nm in diameter [2]. Exosomes contain well-known molecules such as proteins, DNA, RNA, lipids, and metabolites associated with different host cells [3]. Human umbilical cord-derived mesenchymal stem cells (UCMSCs) presenting in the umbilical cord tissue and characterized as self-renewing and multipotent become one of the sources of exosomes [4]. The possibility of noninvasive harvesting, low immunogenicity, and immunoregulatory activity of UCMSCs give them a unique advantage over bone marrow mesenchymal stem cells and adipose mesenchymal stem cells [5]. There is currently no clear report on the long-term safety of MSCs, including UCMSCs, in humans despite various clinical trials and results [6].

On the contrary, exosomes derived from UCMSCs (UCMSC-EVs) are thought to possess the characteristics of UCMSCs that can promote tissue regeneration, improve tissue function [7,8], regulate the immune system [9], and exert anti-inflammatory effects [10]. Many studies have reported the efficacy of UCMSC-EVs in wound healing [11] and bone regeneration [8] by the local administration. However, the biodistribution and pharmacokinetics of UCMSC-EVs in systemic circulation remain unclear. UCMSC-EVs have been labeled with dyes [12], gadolinium [13], or gold nanoparticles [14] and observed by *in vivo* imaging, but UCMSC-EVs labeled and quantified with radioisotopes have not yet been studied. The advantages of radioactive UCMSC-EVs include not significantly altering the structure of exosomes, high sensitivity for capturing regions of interest with three-dimensional images, as well as elucidating pharmacokinetic information [15,16].

Radiolabeling of EVs has been explored for almost a decade since the first report was published in 2015 [17]. Due to similarities in their physical structures, liposome radiolabeling techniques were applied to EVs with minor modifications [18,19]. Technetium-99m (Tc-99m), with a short half-life of 6 h and favorable gamma energy of 140 KeV, is the most common radionuclide used for EV imaging, most likely due to its availability and low cost. Specific EV components, such as histidine and glutathione, are needed for ^99m^Tc-tricarbonyl ([^99m^Tc(CO)_3_]^+^) [20] and ^99m^Tc-hexamethyl propyleneamine oxime ([^99m^Tc]-HMPAO) [21] labeling, respectively. Alternatively, a group reported that they labeled milk-derived EVs with Tc-99m using stannous chloride (SnCl_2_) to reduce unreactive ^99m^Tc^7+^ to ^99m^Tc^4+^. They achieved a radiolabeling yield of 37% with 75 μg of exosomes. However, they observed a slight release of free Tc-99m in the thyroid and stomach [15]. Yet, it is unknown whether Tc-99m-labeled UCMSC-EVs possess improved radiolabeling yield and stability *in vivo*. Herein, our study was the first to evaluate the *in vivo* tracking of exosomes derived from human UCMSCs by single photon emission computed tomography (SPECT) based on labeling with radioactive Tc-99m. The radiolabeled exosomes enabled us to carry out a comprehensive pharmacokinetic assessment of ^99m^Tc-UCMSC-EVs in healthy mice to optimize the dosimetry in further disease treatment applications.

## 2 Materials and methods

### 2.1 UCMSC-EVs preparation

Human UCMSC-EVs were provided by ExoOne Bio Co., Ltd. (Taipei City, Taiwan). The preparation procedure of a previous study was modified [22]. Briefly, human UCMSCs were cultured to 90% confluence in alpha-modified minimum essential medium (αMEM) containing 5% human platelet lysate and maintained in a 10-layered Cell Factory Systems incubator (Cell Factory™ Systems, CF10) at 37 °C and 5% CO_2_. The cell culture medium was harvested and filtered through a 0.22 μm polyether sulfone membrane filter (Thermo Fisher Scientific, Waltham, MA, USA) to remove large particles and cell debris. Finally, EVs were isolated and diafiltrated with 10 x volumes of PBS in a tangential flow filtration (TFF) (Sartorius Stedim Biotech, Göttingen, Germany) system with a 100 kDa molecular weight cutoff filter. The final exosome pellets were stored at −80 °C.

### 2.2 Characterization of UCMSC-EVs: particle size, protein concentration, and protein content

The size distribution and concentration of isolated UCMSC-EVs suspensions were analyzed using nanoparticle tracking analysis (NTA) (NanoSight NS300, Malvern Panalytical Ltd., UK). NTA is widely used in analyzing particle size distribution (nm) and concentration (particles/mL) [23]. NTA analyzes the size of the particles in fluids based on the rate of Brownian motion to dynamic light scattering (DLS) [24]. The samples were diluted with PBS to achieve an optimized systematic measured range of 1 × 10^6^–1 × 10^9^ particles/mL in accordance with the manufacturer's protocol. The final concentration of UCMSC-EVs was calculated by multiplying the measured concentration with the dilution ratio. The protein concentration of EVs was measured using a bicinchoninic acid protein assay (BCA; Thermo Fisher Scientific, Waltham, MA, USA). The measurements of nonlabelled UCMSC-EVs and labeled UCMSC-EVs were performed and compared (n = 3). The UCMSC-EVs were stored at 4 °C with water until their radioactivity decayed, and then the samples were prepared for transmission electron microscopy (TEM). Briefly, the samples were fixed with 3% glutaraldehyde in 0.1 M cacodylate buffer pH 7.4, loaded onto copper-mesh formvar grids, and negatively stained with 4% uranyl acetate for 3 min. Images were acquired using an HT7800 transmission electron microscope at 100 kV (Hitachi, Tokyo, Japan).

### 2.3 Radiolabeling of UCMSC-EVs with ^99m^Tc

**Fig. 1:**
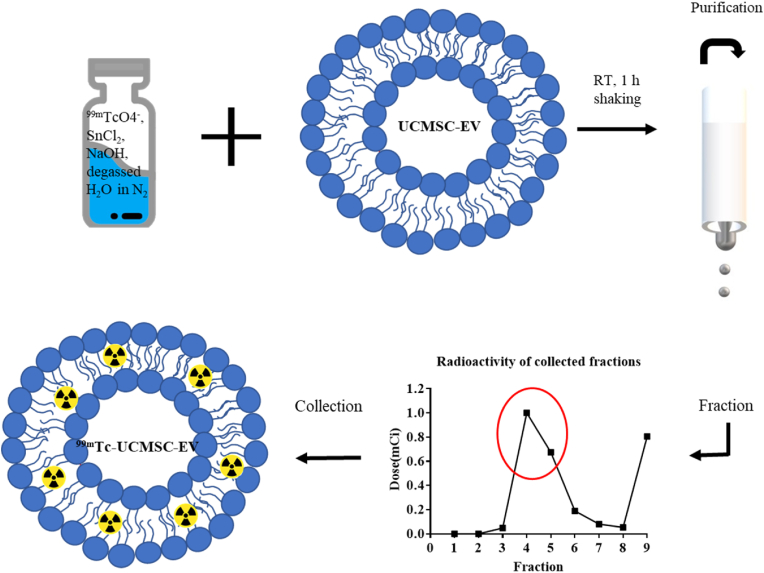
Schematic representation of radiolabeling UCMSC-EVs and purification of ^99m^Tc-UCMSC-EVs with a MiniTrap Sephadex G-25 column. The UCMSC-EVs were labeled with ^99m^TcO_2_, which remained in the hydrophobic form to chelate the phosphonate group of the membrane, also known as surface labeling. The red circle indicates the collected fractions of ^99m^Tc-UCMSC-EVs.

The labeling procedure was performed as previously described by Gonzalez et al. [15] with modifications. Commercial sodium pertechnetate (^99m^Tc-NaTcO_4_) was purchased from Global Medical Solutions (Taiwan, LTD). All reagents were purchased from Sigma‒Aldrich (St. Louis, MO, USA). Briefly, 20 μL of stannous chloride with a concentration of 0.002 M in 10% acetic acid was prepared. An aliquot of 25.5 μL of sodium hydroxide with 2.8 M was added to the prepared stannous chloride solution to adjust the pH to 7–8. The vial with the mixture was degassed in a nitrogen atmosphere for 10 min. An aliquot of 30 μL of ^99m^Tc-NaTcO_4_ with approximately 185 MBq was added into the mixture solution vial and shaken for 5 min. An aliquot of 70 μL of UCMSC-EVs at a concentration of 7.54 × 10^12^ ± 1.03 × 10^12^ particles/mL (protein concentration, 100 μg/100 μL) was added into the Tc-99m reduced vial following the addition of 100–150 μL of degassed water. The final solution was mixed at room temperature for 1 h. The resulting products were purified using a PD MiniTrap Sephadex G-25 column (Cytiva, Wash. D.C., USA) and eluted with degassed water. The fractions of the eluted solution were collected. The radiochemical yield of the reaction was calculated by the ratio of the radioactivity of the purified radiolabeled UCMSC-EVs to the initial loading solution. The radiopurity of the ^99m^Tc-UCMSC-EVs was analyzed using instant thin layer chromatography (iTLC) with normal saline as the mobile phase and a glass microfiber chromatography paper impregnated silica gel (Agilent Technologies, Santa Clara, CA, USA) and measured by a PET/SPECT radio-TLC scanner (LabLogic, Sheffield, UK). Fig. 1 displays the procedures of radiolabeling UCMSC-EVs with Tc-99m and the purification of ^99m^Tc-UCMSC-EVs.

### 2.4 High-performance liquid chromatography (HPLC)

The radiochemical purity of the labeled UCMSC-EVs was evaluated using a Waters™ 600 E High-performance liquid chromatography (HPLC) system (Waters, MA, U.S.A.) equipped with a size exclusion column (Phenomenex, BioSep-SEC-S 3000, 5 μm, 300 × 7.8 mm, plus guard column, U.S.A.). Normal PBS buffer was used as the HPLC mobile phase with a flow rate of 1 mL/min. Data were processed using Waters Empower 2 software. The absorbance at 254 nm and the radioactivity in the eluate were monitored with a PDA W2996 UV detector and a flow count detector (B-FC-1000, Bioscan Inc., Washington DC, U.S.A.), respectively.

### 2.5 In vitro stability studies

The *in vitro* stability of ^99m^Tc-UCMSC-EVs was assessed by incubating 10 μL of ^99m^Tc-UCMSC-EVs in 1X PBS (1:1) with fetal bovine serum (1:1) for 24 h at 37 °C. Two-microliter aliquots of the mixture were analyzed at each time point using iTLC on glass microfiber chromatography paper impregnated with silica gel and normal saline as the mobile phase. A gamma counter (HIDEX, Turku, Finland) was used to measure the radioactivity of the iTLC regions.

### 2.6 Western blot analysis

Fifteen micrograms of total protein from nonlabelled UCMSC-EVs and ^99m^Tc-UCMSC-EVs were separated by SDS-polyacrylamide gel electrophoresis and electrophoretically transferred to a polyvinylidene fluoride (PVDF) membrane (Millipore, MA). PVDF membranes were blocked followed by incubation at 4 °C overnight with the following primary antibodies diluted in PBST: CD 9 (1:1000, Cell Signaling Technology, Danvers MA, USA), CD 63 (1:1000, Abcam, Waltham, MA, USA), CD81 (1:1000, System Biosciences, Palo Alto, CA, USA), CD73 (1:1000 Proteintech, Chicago, IL, USA), HSP70 (1:1000, Cell Signaling Technology, Danvers MA, USA), Alix (1:1000, Cell Signaling Technology, Danvers MA, USA), and TSG101 (1:1000, Cell Signaling Technology, Danvers MA, USA) as well as the negative control markers calnexin (1:1000, Abcam, Waltham, MA, USA) and β-actin (1:10,000, Abcam, Waltham, MA, USA). A subsequent incubation with a secondary antibody (1:10,000) was conducted at room temperature for 2 h, and immunochemical detection was performed using a chemiluminescence ECL detection system (GE Healthcare, South Jakarta, Indonesia).

### 2.7 Ethics statement

The animal experiments complied with the AAALAC guidelines for animal experiments. The Chang Gung Memorial Hospital Animal Care and Use Committee approved all the procedures (IACUC 2022052601).

### 2.8 In vivo SPECT/CT imaging

The SPECT and computed tomography (CT) scans were acquired from healthy BALB/c mice (8–14 weeks old, 21–30 g in weight, n = 3 for male, n = 3 for female) after administration of the ^99m^Tc-UCMSC-EVs by intravenous tail injection (100 μL, 7.3 ± 0.57 MBq, ∼1.03 × 10^11^ particles/ml). ^99m^Tc-NaTcO_4_ was used as a control (100 μL, 18.5 MBq, n = 1). Images were acquired with the animals under 2% isoflurane anesthesia. Longitudinal *in vivo* tracking of ^99m^Tc-UCMSC-EVs was carried out using a small animal imaging scanner SPECT/CT (nanoScan, Mediso, Hungary). The animals were placed in the prone position, and the field of view was adjusted to the whole body of the mouse. Images were acquired at 30 min, 1 h, 4 h, and 24 h postinjection. The 30-min scan time was performed for each experiment except 60-min scan time for 24 h time-point. Multipinhole collimators were used for the acquisition of the SPECT images with a 20% energy window and 0.85 mm spatial resolution. The 3D OSEM reconstruction was performed with a 0.26 mm^3^ voxel size. The SPECT image was subjected to attenuation, scatter correction, and isotope decay correction. For the acquisition of anatomical images, the selected CT parameters were 50 KeV, 170 μA, 360 projections, and 1:4 binning. CT images were reconstructed with a 0.25 mm^3^ voxel size. The regions of interest (ROIs) were semiautomatically determined using a threshold of 20% of the maximum %ID/g minus the minimum %ID/g from manually contoured regions. ^99m^Tc-UCMSC-EVs uptake in regions was expressed as the mean of %ID/g. All images were analyzed using PMOD version 4.1 (PMOD Technologies LLC, Fällanden, Switzerland).

### 2.9 Pharmacokinetics of ^99m^Tc-UCMSC-EVs

The half-life of ^99m^Tc-UCMSC-EVs in the blood was determined by measuring radioactivity in serial blood samples at 2 min, 5 min, 10 min, 20 min, 30 min, 1 h, 4 h, and 24 h. The mice were also subjected to SPECT scans to study the pharmacokinetics of ^99m^Tc-UCMSC-EVs. Blood samples (5 μL) were collected from the tail vein of mice under 2% isoflurane anesthesia at several time points postinjection. The radioactivity of blood samples was measured using a Wallac Wizard 1480 Automatic Gamma Counter (PerkinElmer, Waltham, MA, USA). The measurements in counts per minute of the blood samples were corrected to whole-body blood volume and normalized to the percentage injection dose. The density of blood is 1.05 g/cm^3^, and the weight of blood is 8% of the body weight. The volume of the whole-body blood was calculated by multiplying the mouse body weight by 8% and dividing that by 1.05 g/cm^3^. Data were analyzed with a two-phase decay nonlinear regression in GraphPad Prism 6.0 (GraphPad Software, La Jolla, CA, USA). The blood half-life of ^99m^Tc-UCMSC-EVs in the distribution phase and elimination phase was computed (n = 6). The area under the curve (AUC) of ^99m^Tc-UCMSC-EVs was calculated by radioactivity of blood per mL (n = 6).

### 2.10 Ex vivo biodistribution studies

Biodistribution experiments were conducted on healthy BALB/c mice (8–14 weeks old, 21–30 g in weight, n = 3 for male, n = 3 for female) after administration of the ^99m^Tc-UCMSC-EVs by intravenous tail injection (50 μL, 0.75 ± 0.05 MBq, ∼2.06 × 10^10^ particles/ml). Mice were sacrificed 30 min, 1 h, 4 h, and 24 h postinjection, and their organs were harvested (i.e., blood, brain, trachea/thyroid, heart, lungs, liver, spleen, stomach, kidneys, intestines, muscle, bone, skin, and tail). The injection dose was corrected by deducting the radioactivity of the tail. The radioactivity in the tissue of interest was measured using a Wallac Wizard 1480 Automatic Gamma Counter and expressed as % ID/g.

### 2.11 Histological analysis and autoradiography

To investigate the expression of ^99m^Tc-UCMSC-EVs in the tissue, immunofluorescence staining (IF) and autoradiography (ARG) were conducted to verify the distribution of ^99m^Tc-UCMSC-EVs in the high uptake organ, the spleen. Mouse spleens were excised after injection of 7.4 MBq of ^99m^Tc-UCMSC-EVs for 24 h, and spleen tissues were embedded in OCT compound (Leica), followed by cryosectioning with a thickness of 20 μm. The autoradiogram images were captured using a phosphor image reader (FLA-5100; Fujifilm, Tokyo, Japan) with an exposure time of 72 h. None of the ^99m^Tc-UCMSC-EVs were used as a control group.

After the radioactivity in the tissues had decayed thoroughly, the slides were subjected to IF staining procedures following the manufacturer's protocol. Briefly, the sections were first fixed with ice-cold acetone for 5 min, air dried at room temperature for 30 min, and quickly rinsed with ice-cold PBS (1X) for 3 min. The nonspecific binding signal was blocked with ice-cold 5% BSA in goat serum for 1 h. In the next step, the sections were incubated with an anti-CD73 antibody (Proteintech, Chicago, IL, USA) at a dilution of 1:200 for 1 h at room temperature. After incubation with the primary antibody, the sections were washed with ice-cold PBST with 0.2% Tween-20 for 5 min in triplicate. Then, the sections were incubated with fluorescent dye-conjugated secondary antibody (ab150077, Abcam, MA, USA) for 1 h at room temperature and quickly rinsed with ice-cold PBS. Coverslips were mounted with mounting medium, and nuclei were counterstained with DAPI. Images were captured using a HistoFAXS Tissue Analysis System (TissueGnostics, Wien, Austria).

To study the impact of ^99m^Tc-UCMSC-EVs on liver accumulation, Western blot techniques were utilized on mouse liver tissues. After administering 7.4 MBq of ^99m^Tc-UCMSC-EVs for 24 h, liver tissues were collected, homogenized, and lysed with RIPA. Control group mice were not given ^99m^Tc-UCMSC-EVs. The liver proteins were extracted and analyzed via Western blot to determine the expression of CD9 and CD63 proteins (as described in section 2.6).

### 2.12 Statistical analysis

The results are expressed as the mean ± standard deviation (SD). Data were processed using GraphPad Prism 6.0. ANOVA was used for the statistical analyses of ^99m^Tc-UCMSC-EVs in each organ at different time points, as well as their stability *in vitro*. The Western blot statistical analysis was used to compare the control group with the group that received ^99m^Tc-UCMSC-EVs administration using *t*-test and nonparametric tests with two tails.

## 3 Results

### 3.1 Radiolabeled ^99m^Tc-UCMSC-EVs

**Fig. 2:**
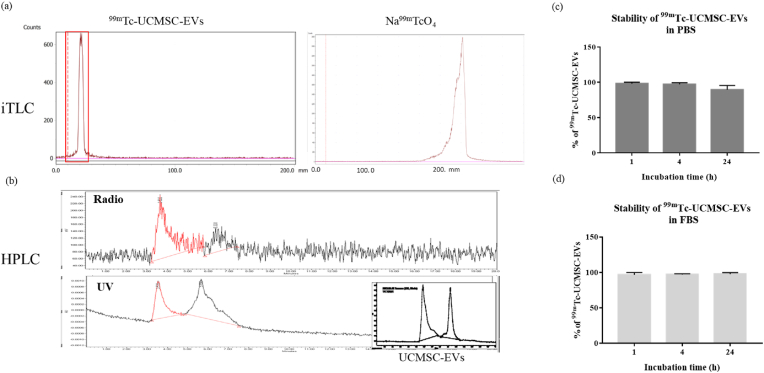
Analysis of ^99m^Tc-UCMSC-EVs. (a) The radiochemical purifications of ^99m^Tc-UCMSC-EVs and ^99m^Tc-NaTcO4 were determined by iTLC on glass microfiber chromatography paper impregnated with silica gel and normal saline as the mobile phase. A purity of >99.9% of ^99m^Tc-UCMSC-EVs was achieved before the animal study. (b) Radioactive and UV HPLC chromatograms of ^99m^Tc-UCMSC-EVs were obtained. The radioactive and UV chromatogram peaks representing ^99m^Tc-UCMSC-EVs showed the same retention time. (c, d) The stability of ^99m^Tc-UCMSC-Evs was examined after incubation with PBS and FBS for 1 h, 4 h, and 24 h at 37 °C. Slightly decreased stability of ^99m^Tc-UCMSC-EVs at 24 h after incubation with PBS was found.

The purity of the ^99m^Tc-UCMSC-EVs was determined using iTLC [Fig. 2a], which confirmed a radiopurity >99% after purification. The main peak in the radioactive HPLC chromatogram of ^99m^Tc-UCMSC-EVs was observed at 3.63 min, matching the retention time of pure nonlabelled UCMSC-EVs in the UV chromatogram (254 nm) [Fig. 2b]. The radiochemical yield of ^99m^Tc-UCMSC-EVs was 52.2 ± 3.59% (n = 3). The stability of ^99m^Tc-UCMSC-EVs was 98.80 ± 0.22% and 89.90 ± 2.08% at 1 h and 24 h with PBS incubation [Fig. 2c] and 96.76 ± 3.29% and 97.82 ± 2.08% at 1 h and 24 h with FBS incubation (n = 3 for each group) [Fig. 2d].

### 3.2 Characterization of UCMSC-EVs after radiolabeling

**Fig. 3:**
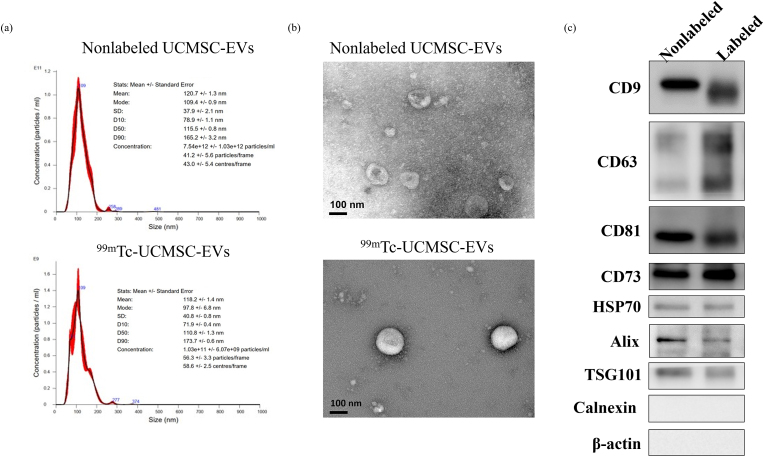
Characterization of UCMSC-EVs and ^99m^Tc-UCMSC-EVs. (a) Particle size analysis and (b) TEM of nonlabelled UCMSC-EVs and ^99m^Tc-UCMSC-EVs. There was no significant change in the particle size and morphology of UCMSC-EVs between prelabeling and postlabeling with ^99m^Tc. (c) Western blot analysis for exosome marker proteins of nonlabelled UCMSC-EVs and ^99m^Tc-UCMSC-EVs (labeled). Slightly decreased expression levels of the exosome marker proteins were observed in ^99m^Tc-UCMSC-EVs.

The protein concentration of UCMSC-EVs was equivalent to approximately 118.8–287.8 μg/100 μL. Consistently, the NTA analysis confirmed a hydrodynamic particle size of 116.9 ± 7.29 nm for nonlabelled UCMSC-EVs and 129.7 ± 10.51 nm for radiolabeled UCMSC-EVs with triplicate samples. The size of UCMSC-EVs did not show significant changes after labeling [Fig. 3a and b]. To assess changes in the exosome-specific protein contents of UCMSC-EVs after radiolabeling, the expression of exosomal markers CD9, CD63, CD81, CD73, HSP70, Alix, and TSG101 was detected by Western blot analysis. Visually, the expression of the exosomal-specific proteins was not significantly altered after radiolabeling [Fig. 3c].

### 3.3 In vivo SPECT/CT imaging

**Fig. 4:**
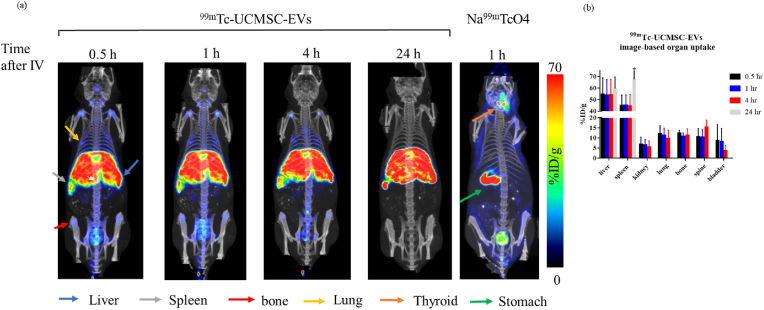
Assessment of the intravenous administration of ^99m^Tc-UCMSC-EVs and comparison with ^99m^Tc-NaTcO4. (a) *In vivo* SPECT/CT fused representative images of ^99m^Tc-UCMSC-EVs and Na^99m^TcO4 at various time points postinjection. Significant uptake of ^99m^Tc-UCMSC-Evs was found in the liver, spleen, lung, and bone. The elimination route of ^99m^Tc-UCMSC-EVs was shown to be through the urinary tract. Furthermore, there was no observed uptake in the thyroid and stomach for the ^99m^Tc-UCMSC-EVs group compared with the Na^99m^TcO4 group, indicating that the ^99m^Tc-UCMSC-EVs were stable *in vivo*. (b) Quantification of ^99m^Tc-UCMSC-EVs in organs/tissues of interest at various time points postinjection. The spleen uptake of ^99m^Tc-UCMSC-EVs 24 h postinjection was significantly elevated compared with that at previous time points (67.57 ± 10.65%ID/g, ∗). No ^99m^Tc-UCMSC-EVs were observed in the kidney, lung, bone, spine, or bladder. ∗, p < 0.05.

Small animal SPECT/CT studies were carried out after administering ^99m^Tc-UCMSC-EVs to healthy mice. Substantial EV accumulation was observed in the bladder within 30 min postinjection, which confirms EV excretion through the urinary tract (bladder activity: 8.71 ± 7.84 %ID/g). Mainly, ^99m^Tc-UCMSC-EVs uptake in the spleen (44.82 ± 9.19%ID/g) and liver (54.65 ± 14.18 %ID/g) occurred after 30 min, and no significant changes in the ^99m^Tc-UCMSC-EVs distribution were observed at further time points, except in the spleen at 24 h (67.57 ± 10.65 %ID/g, p < 0.05). Notably, ^99m^Tc-UCMSC-EVs uptake in the kidneys, lungs, bone, and spine was not observed at 24 h. Significantly high uptake of ^99m^TcO_4_^-^ in the thyroid and stomach was observed in a control mouse [Fig. 4].

### 3.4 Pharmacokinetics of ^99m^Tc-UCMSC-EVs

**Fig. 5:**
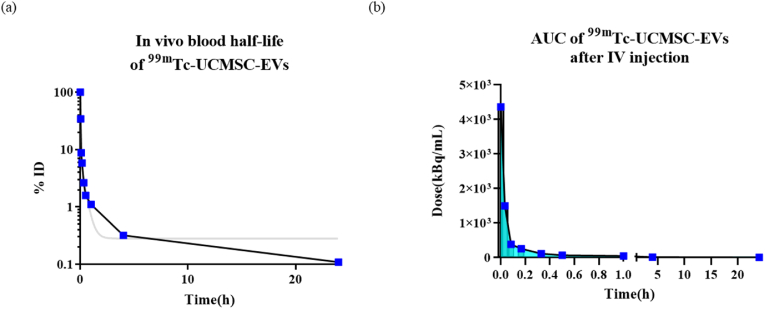
Pharmacokinetics of ^99m^Tc-UCMSC-EVs (a) The representation of *in vivo* blood half-life fitting with a two-compartment model. The gray line is the fitting curve. The blood half-life was 0.85 ± 0.28 min in the distribution phase and 25.22 ± 20.76 min in the elimination phase (n = 6). (b) A representative plot of the AUC of ^99m^Tc-UCMSC-EVs is shown from the initial timepoint to 24 h postinjection. Collectively, the AUC of ^99m^Tc-UCMSC-EVs in blood was 1021.07 ± 397.77 kBq∗h/mL from administration to 24 h (n = 6).

The *in vivo* blood half-life of ^99m^Tc-UCMSC-EVs showed a quick distribution and moderate elimination in the two-compartment model [Fig. 5]. The blood half-life was 0.85 ± 0.28 min for the distribution phase and 25.22 ± 20.76 min for the elimination phase. The AUC of ^99m^Tc-UCMSC-EVs in blood was 1021.07 ± 397.77 kBq∗h/mL from administration to 24 h.

### 3.5 Ex vivo biodistribution studies

Biological distribution was performed at four different time points (30 min, 1, 4, and 24 h postinjection) in healthy BALB/c mice. The distribution of ^99m^Tc-UCMSC-EVs in tissues *ex vivo* was consistent with that of ^99m^Tc-UCMSC-EVs in tissues *in vivo*. The main portion of ^99m^Tc-UCMSC-EVs accumulated in the spleen and liver, while other organs, including the lung, kidney, bone, and thyroid, showed moderate levels of radioactivity. The brain, stomach, intestines, and muscle showed very low levels of radioactivity from 30 min to 24 h [Table 1].

**Table 1:** Biodistribution of^99m^Tc-UCMSC-EVs at 0.5 h, 1 h, 4 h and 24 h after intravenous administration.

Organ (mean ± std %ID/g, n = 6)	0.5hr	1hr	4hr	24hr
blood	1.61 ± 0.89	0.89 ± 0.66	0.37 ± 0.27	0.13 ± 0.03
brain	0.04 ± 0.03	0.03 ± 0.02	0.01 ± 0.01	0.01 ± 0.00
thyroid	1.31 ± 0.64	1.29 ± 0.65	1.06 ± 0.53	0.39 ± 0.21
heart	0.57 ± 0.26	0.34 ± 0.21	0.22 ± 0.17	0.15 ± 0.06
lung	3.97 ± 3.32	2.52 ± 1.62	5.95 ± 6.14	4.55 ± 6.95
liver	64.74 ± 39.99	63.06 ± 33.71	63.03 ± 37.54	53.60 ± 30.85
stomach	0.47 ± 0.19	0.46 ± 0.19	0.27 ± 0.07	0.15 ± 0.06
pancrease	0.22 ± 0.09	0.21 ± 0.21	0.12 ± 0.06	0.05 ± 0.02
spleen	75.68 ± 47.34	81.95 ± 50.92	63.94 ± 27.48	86.09 ± 38.19
intestine	0.45 ± 0.15	0.30 ± 0.15	0.18 ± 0.11	0.14 ± 0.04
kidney	3.66 ± 1.49	2.85 ± 1.3	2.13 ± 0.89	1.61 ± 0.25
skin	0.34 ± 0.25	0.20 ± 0.12	0.13 ± 0.05	0.12 ± 0.03
muscle	0.07 ± 0.03	0.06 ± 0.03	0.04 ± 0.03	0.02 ± 0.05
bone	3.71 ± 1.97	3.08 ± 1.62	3.21 ± 2.28	2.84 ± 0.88

### 3.6 Histological analysis of ^99m^Tc-UCMSC-EVs in spleen

**Fig. 6:**
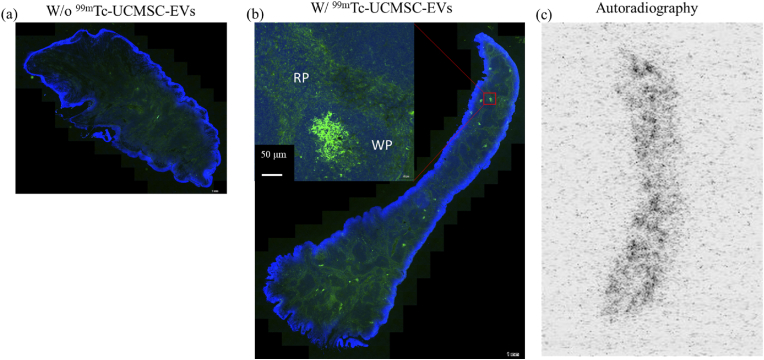
*Ex vivo*^99m^Tc-UCMSC-EVs distribution in spleen tissues. The expression of CD73 was unevenly distributed in the spleen. Uniform expression was found in the red pulp and concentrated expression was found in the white pulp. (a) Control mouse without ^99m^Tc-UCMSC-EVs injection. (b) The upregulated expression of CD73 and DAPI in the spleen of the ^99m^Tc-UCMSC-EVs-treated mouse. (c) The corresponding concentrated radioactivity signal by autoradiography in the spleen of the ^99m^Tc-UCMSC-EVs-treated mouse.

**Fig. 7:**
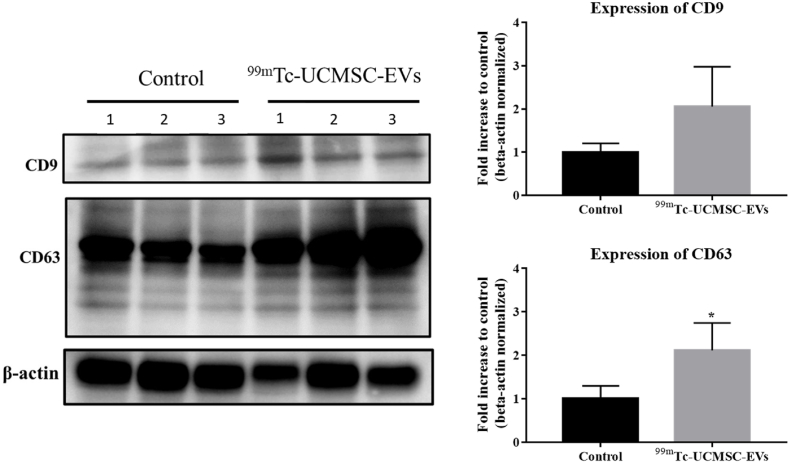
The protein expression of *ex vivo*^99m^Tc-UCMSC-EVs in liver tissues was measured quantitatively. A Western blot analysis was conducted to identify exosome marker proteins in both the ^99m^Tc-UCMSC-EVs-treated group and the untreated control group. The results showed a significant increase in the expression levels of CD63 in ^99m^Tc-UCMSC-EVs (fold increase to control with beta-actin normalized, 2.11 ± 0.63, n = 3, ∗) compared to the control (1 ± 0.30, n = 3). ∗, p < 0.05.

The expression of CD73 on EVs in spleen tissue 24 h after administration of ^99m^Tc-UCMSC-EVs is shown. The control group displayed much lower CD73 expression levels. Notably, the distribution of EVs is uneven due to heterogeneity in the spleen tissues. There was uniform expression in the red pulp and concentrated CD73 expression in the white pulp of spleen tissues. The autoradiogram image results demonstrated this heterogeneity of the ^99m^Tc-UCMSC-EVs signal, which was in accordance with the IF image [Fig. 6]. Moreover, Supplemental data 1 presents CD63 expression in liver and spleen tissues and CD73 expression in liver tissues of control and ^99m^Tc-UCMSC-EVs administration groups. Quantitative Western blot analysis revealed the presence of CD9 and CD63 on EVs in liver tissue 24 h after administering ^99m^Tc-UCMSC-EVs. The ^99m^Tc-UCMSC-EVs group showed significantly higher CD63 expression (fold increase to control with beta-actin normalized, 2.11 ± 0.63, n = 3, p < 0.05) compared to the control group (1 ± 0.30, n = 3), but no significant changes in CD9 expression (fold increase to control with beta-actin normalized, 2.06 ± 0.92, n = 3, p = 0.2) compared to the control group (1.00 ± 0.2, n = 3) [Fig. 7].

## 4 Discussion

UCMSC-EVs have been used in the treatment of several diseases [22,25,26]. The biodistribution and pharmacokinetics of radiolabeled UCMSC-EVs via intravenous administration were examined in our study, which could be used to investigate the potential indications or adjust the current use. Our study is the first to use direct labeling of UCMSC-EVs with the radioisotope Tc-99m. The ^99m^Tc-UCMSC-EVs radiochemical yield was 52 %, and purity was greater than 99 % after purification. The characterization of ^99m^Tc-UCMSC-EVs was not altered compared with nonlabelled UCMSC-EVs. We found that ^99m^Tc-UCMSC-EVs remained stable for 24 h during incubation with FBS, while dissociation of only ^99m^TcO_4_^-^ from UCMSC-EVs occurred during incubation with PBS. Furthermore, the high stability of ^99m^Tc-UCMSC-EVs was verified by SPECT images and biodistribution analyses because of the less uptake in the thyroid and stomach. Upon performing a histological analysis on *ex vivo* liver and spleen tissues of mice, it was observed that the specific proteins found on UCMSC-EVs remained present. Hence, we conducted a thorough study on the stability, distribution, and accumulation of UCMSC-EVs in small animals using radiolabeled Tc-99m.

In previous studies [21,27], exosomes were radiolabeled with ^99m^Tc-HMPAO or ^99m^Tc-tricarbonyl. However, the specific molecular contents of exosomes can interfere with chelating labeling. For example, glutathione (GSH) converts ^99m^Tc-HMPAO from the hydroponic form to the hydrophilic form. This conversion results in the entrapment of ^99m^Tc-HMPAO inside the exosomes [21]. In contrast, labeling exosomes with ^99m^Tc-tricarbonyl showed high yield and suitable stability without purification by interacting with the designed ankyrin repeat proteins on the surface of exosomes [20]. In our experiment, we modified the ^99m^Tc direct labeling strategy that was published by Gonzalez et al. [15]. Wemade sure to adjust the pH value of the SnCl_2_ solution to 7–8 before adding ^99m^TcO4^-^ and UCMSC-EVs. This ensured that the conditions were neutralized, which was stabilized during the labeling process. Furthermore, to improve the labeling efficacy of ^99m^Tc-UCMSC-EVs, we implemented higher reaction volumes of 300 μL to reduce vial wall sticking and longer incubation time compared with the previous report. Additionally, a Sephadex G-25 column and Minitrap to replace the Amicon Ultra 0.5 filter was employed for purification. The new centrifugal filter improved the recovery rate of ^99m^Tc-UCMSC-EVs. The labeling yield achieved 52 % with >99 % radiochemical purity. However, the ^99m^Tc-UCMSC-EVs imaging approach may underestimate the efficacy of UCMSC-EVs if the dosage is translating to future treatment use. This is because the injection of ^99m^Tc-UCMSC-EVs *in vivo* study, including unlabeled UCMSC-EVs, presents a hurdle.

^99m^Tc-UCMSC-EVs administered intravenously showed predominantly hepatic uptake, most likely by Kupffer cells. Similarly, this study showed that the exosomes were mainly taken up by the liver and spleen, which is in line with previous research [27]. However, we found substantial ^99m^Tc-UCMSC-EVs uptake in the lung, bone marrow, and spine, which may lead to a hypothesis of homing effect. In terms of the clinical application and mechanisms of action of UCMSC-EVs, UCMSC-EVs have been used in the treatment of respiratory diseases [28], arthritis [29], and spinal cord injury [30]. Less radioactivity in the thyroid and stomach after intravenous administration was observed in the biodistribution results, indicating the high stability of ^99m^Tc-UCMSC-EVs *in vivo*. Only a small amount of dissociated ^99m^TcO_4_^-^ was taken up through the sodium-dependent iodide transporter NIS [31]. In a pharmacokinetic experiment, the fast blood distribution (0.85 ± 0.28 min) and elimination time (25.22 ± 20.76 min) of ^99m^Tc-UCMSC-EVs were calculated. González et al. reported a blood half-life of 3.84 min after tail vain administration of ^99m^Tc-milk-derived EVs [15], but Lai et al. showed a distribution phase of 19.9 min followed by an elimination phase with a longer half-life of 184.5 min after intravenous administration of EV-GlucB [32]. It seems that the variable of blood half-life of EVs depends on the types of EVs, the fitting mathematical models, as well as the administration routes.

In previous studies, Li et al. discovered that UCMSC-EVs have anti-inflammatory properties that can help regulate allergies [33]. They also found that these EVs can induce antioxidant defense systems in HaCaT cells to serve as an anti-aging component for skin care [34], according to *in vitro* studies. Comprehensive studies have shown that neuroinflammation plays a crucial role in the pathogenesis of neurodegenerative diseases such as Alzheimer's disease and Parkinson's disease [10,35]. Building on these findings, our future research will focus on *in vivo* studies related to neurodegenerative diseases to explore the potential use of UCMSC-EVs as a treatment option. However, compared to milk-derived exosomes [15], we found that ^99m^Tc-UCMSC-EVs did not show significant uptake in the brain in an LPS-induced mouse via intravenous administration or in a healthy mouse via intranasal administration (data not shown). Brain endothelial cell-specific EVs have been demonstrated to be capable of crossing the blood‒brain barrier to transport molecular signals [36]. However, it seems that the origin of ^99m^Tc-UCMSC-EVs could prevent them from crossing the blood‒brain barrier. Focus ultrasound has been applied to enhance the delivery of intravenously administered exosomes by temporarily opening the blood‒brain barrier [37]. Therefore, through UCMSC-EVs combined with focused ultrasound techniques to mediate neuroinflammation, patients with neurodegenerative diseases may have more positive outcomes.

## 5 Conclusion

UCMSC-EVs have moderate radiochemical yield with Tc-99m sodium pertechnetate surface labeling. ^99m^Tc-UCMSC-EVs showed extremely high stability *in vitro* and *in vivo*. The uptake of ^99m^Tc-UCMSC-EVs after intravenous administration was primarily in the liver and spleen, with minor uptake in the lungs, bone, and spine. The ^99m^Tc-UCMSC-EVs circulated in the blood circulation with a half-life of 25 min and were eliminated through the urinary tract, as demonstrated by SPECT imaging, biodistribution analyses, and pharmacokinetics. UCMSC-EVs are expected to be useful in various biomedical applications in the future, thanks to the results of radiolabeling and studying their biodistribution and pharmacokinetics.

## Funding

This research and the APC were funded by ExoOne Bio Co., Ltd., the National Science and Technology Council, Taiwan (Grant 10.13039/501100004663MOST 111-2314-B-182A-011-MY2 for YHC, 10.13039/501100004663MOST 110-2314-B-182-029- for CCW, 10.13039/501100004663MOST 111-2623-E-182-004-NU for CCW, and 10.13039/501100004663MOST 112-2623-E-182-001-NU for CCW), the grants from the Research Fund of 10.13039/100012553Chang Gung Memorial Hospital (CMRPG3N0461 for YHC, CMRPD1M0241 for CCW and CMRPD1M0242 for CCW), and the grant from the Healthy Aging Research Center, 10.13039/501100002836Chang Gung University, Taoyuan, Taiwan (URRPD1P0201).

## Conflict of interest disclosure

The authors Wei-Cheng Tsai and Zhi-Xiang Li were employed by the company ExoOne Bio Co., Ltd. The remaining authors declare that the research was conducted in the absence of any commercial or financial relationships that could be construed as a potential conflict of interest.
