# Supplementary material for: In vivo SPECT imaging of Tc-99m radiolabeled exosomes from human umbilical-cord derived mesenchymal stem cells in small animals
Source: Biomed J. 2024 Apr 16;47(5):100721. doi: 10.1016/j.bj.2024.100721 (PMC11401219; doi:10.1016/j.bj.2024.100721)

Supplemental data 1 presented the immunofluorescent staining of liver and spleen tissues with CD63 and CD73 antibodies.


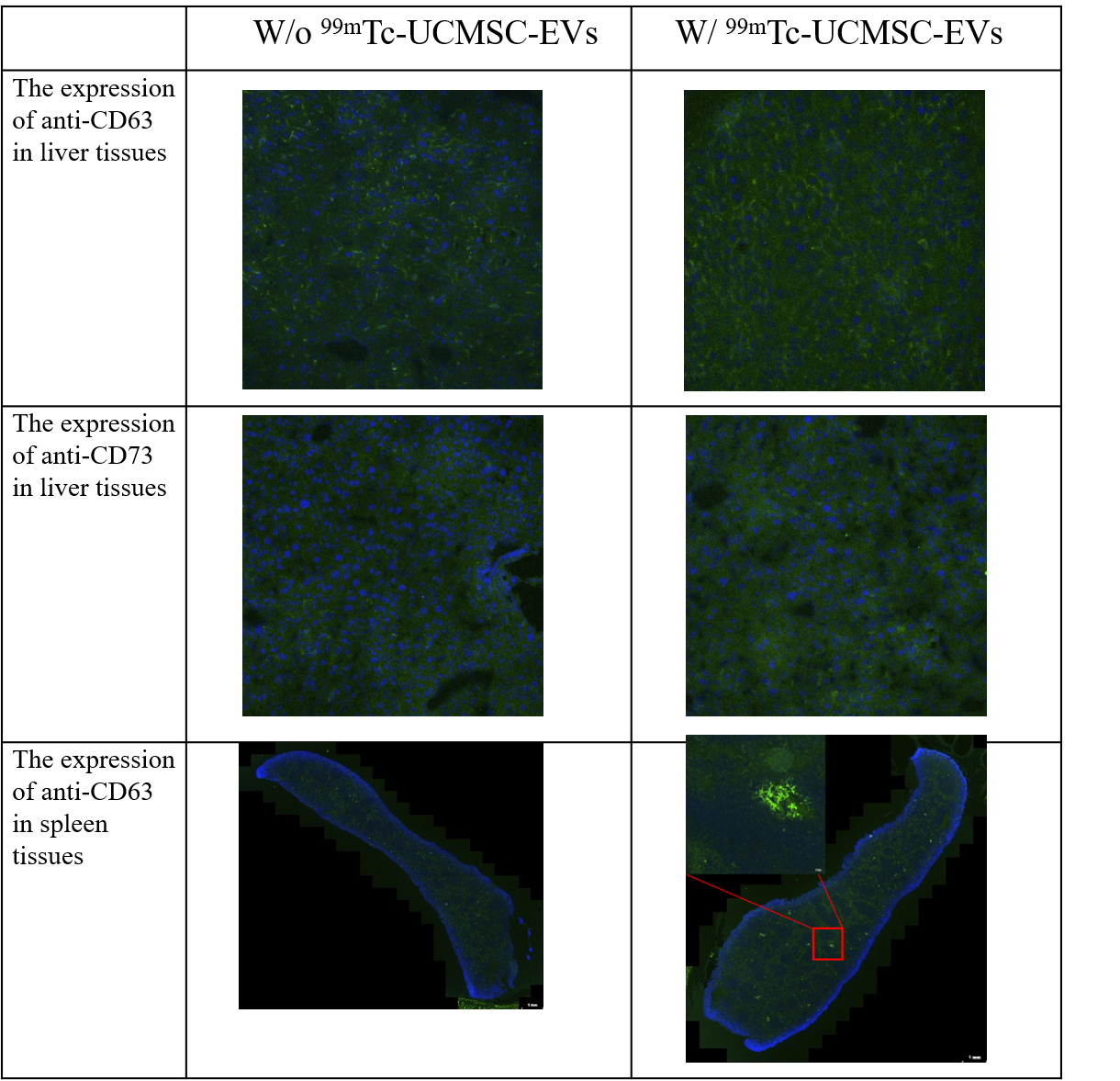

Supplement: Multimedia component 1 [file mmc1.docx]
